# Reengineering the Concept of Emergency Care

**DOI:** 10.1111/acem.70424

**Published:** 2026-10-09

**Authors:** R. Andrew Taylor

**Affiliations:** ^1^ Department of Emergency Medicine University of Virginia Charlottesville Virginia USA

## Abstract

Emergency departments (EDs) absorb most acute unscheduled care (AUC) in the United States. Routing all of this care through one high‐intensity system serves no one well: crowding delays care for time‐critical patients; visits that turn on evaluation and coordination, and routine and chronic care defined by longitudinal needs, are delivered episodically and at high cost in a setting built for resuscitation and stabilization; and clinicians trained for emergencies spend much of their time on work the category does not fit. I argue that this is not only a capacity or workflow problem but a conceptual one. In practice, “emergency care” is defined by arrival: crossing the ED threshold, not clinical risk, triggers the legal duties, insurance protections, and workflows attached to the label. This was no one's mistake; routing everything through the one door always open was a rational adaptation to a fragmented system. But the adaptation no longer merely persists; it forecloses alternatives. Using conceptual engineering, I trace how history, regulation, payment, public scripts, and lexical entrenchment stabilize this system, and situate it against the social drivers that make ED‐as‐default ethically necessary and operationally costly. I then propose an ameliorated concept of emergency care: a risk‐ and time‐sensitive, expert‐mediated, priority‐conferring subset of AUC, determined by structured triage wherever it occurs. Because a definition changes nothing by itself, I outline a phased implementation pathway, show how it applies across delivery settings, including rural and safety‐net systems where the ED should remain the local acute care hub, and describe the workforce implications for emergency medicine. The choice posed to the specialty is whether to keep treating crowding, mismatched care, and clinician dissonance as operational problems to optimize within the ED, or to recognize them as properties of what we mean by emergency care and of the system that enacts it.

## 1 Introduction

An older adult on warfarin has her INR checked by an outside lab. It is markedly elevated. She has no bleeding and no other symptoms, and no anticoagulation clinic appointment for a week. What she needs is a same‐day laboratory check, a dosing decision, medication reconciliation, and a scheduled recheck. She is sent to the emergency department (ED) because nowhere else can do all four today. This is not an emergency, but it cannot safely wait a week either. Her arrival, not her risk, classifies the encounter, activating the legal duties, coverage entitlements, and institutional expectations reserved for emergencies. The label says rescue; the work is adjustment and arrangement.

EDs are built for resuscitation and stabilization: sudden, life‐threatening conditions met with speed and precision [1]. In practice they absorb far more, including chronic disease flares in patients without primary care access, undifferentiated symptoms whose seriousness becomes apparent only after evaluation, and coordination‐heavy problems like the INR case [2, 3]. As the universal intake channel for acute unscheduled care (AUC), the ED pools presentations that differ enormously in the downside of delay and in the coordination they require, then runs all of them through one high‐intensity workflow and labels the entire output “emergency care.” The result is a lose–lose: crowding‐driven delays risk harm to the time‐critical, while lower‐risk, coordination‐heavy visits are managed in a setting poorly matched to their needs.

None of this is news to emergency clinicians, who know the ED is not the same thing as emergency care. What is missing is an account of why decades of throughput and triage reform have changed so little. Those reforms improved how the ED processes what arrives. None touched what arrives, or why: legal duties, coverage and payment rules, and institutional workflows still attach the “emergency” label at the ED door rather than at the moment of structured risk assessment, and any intervention that leaves that attachment intact can make the sorting faster but not different.

This is not an error anyone committed. The ED became the default because it is the one institution that must take all comers, at any hour, whatever their ability to pay—a social welfare role of last resort [4, 5]. Sending the anticoagulated patient there was locally rational; so is every other referral like it. But an adaptation repeated for decades stops being a response and becomes the architecture. The concept of emergency care is part of what holds this up, and that makes it a target for repair.

Conceptual engineering [6, 7] offers a method for this repair, and the paper makes four contributions. First, it diagnoses the dysfunction as a conferral problem: the institutional label “emergency” is triggered by arrival, not by risk. Second, it locates a bundling error in which clinical classification, resource allocation, and legal or payment designation collapse into that single location‐based trigger. Third, it proposes a replacement concept: emergency care as a risk‐ and time‐sensitive, expert‐mediated, priority‐conferring subset of AUC, conferred by structured triage wherever it occurs. Fourth, it sets out a phased implementation pathway, shows how that pathway changes across delivery settings from integrated systems to rural and safety‐net EDs, and draws out the workforce implications for emergency medicine. This is a normative conceptual analysis, not a systematic review; it draws on professional definitions, regulatory texts, and the conceptual engineering literature. A Supplement provides extended hypothetical objections and replies (Data S1–S6) on safety, paternalism, equity, feasibility, the “capacity‐not‐concepts” critique, and the ED's safety‐net role.

## 2 Target Concepts and Problem Statement

The term *emergency* is used in multiple, often conflated ways: a property of a clinical condition (“this is an emergency”), a kind of service (“emergency care”), a medical specialty (“emergency medicine”), and a physical site (“the emergency department”). For analytic purposes, I distinguish:

**Emergency (clinical state)**. A condition in which, on the information available at the time of assessment, failure to intervene within a short time horizon carries a substantial risk of serious harm or death.

**Emergency care (service concept)**. A set of time‐sensitive interventions and decisions organized to address such conditions, with priority access to constrained resources.

**Emergency departments (institutional sites)**. Hospital‐based facilities equipped and staffed for continuous unscheduled evaluation, historically the main locations where emergency care has been concentrated, are now also sites for a wide spectrum of AUC.

**Acute unscheduled care (AUC)**. The broader class of time‐sensitive or distressing health problems arising without advance scheduling, ranging from trivial to life‐threatening.

The target of conceptual engineering in this paper is *emergency care* as a service concept: the normative category that should guide the design of institutions, triage processes, and professional roles. I treat *emergency* as the associated classificatory concept for clinical states that warrant such care, and *emergency departments* as one, but not the only, site where emergency care can be provided.

The central claim is that, in many U.S. settings, emergency care is institutionally and publicly assigned through an ED‐centered conferral architecture rather than identified through risk‐ and time‐sensitive assessment, making ED presentation a poor proxy for emergency status. Three consequences follow: the ED is overextended as a catch‐all access point; clinicians trained for high‐acuity, time‐critical care carry caseloads the training does not fit; and public and institutional expectations cast the ED as the default remedy for system failures, expanding its mandate without commensurate alternative AUC capacity. A concept that spans cardiac arrest, ankle sprains, and supratherapeutic INR assessments cannot tell an institution what to prioritize, a clinician what role to occupy, or a system what to build.

## 3 Conceptual Engineering Framework

A concept does more than label. It shapes which problems are visible, which solutions seem legitimate, and how institutions, metrics, and laws organize themselves around it [8]. Conceptual engineering asks whether a concept, in its current form, is fit for the purposes it needs to serve, and how it might be revised when it is not [9]. Two ideas from this literature matter here. The first is *amelioration*: when a concept is failing the work asked of it, the appropriate response is to propose a revised version that does that work better, judged against explicit design criteria. Haslanger's treatment of social categories is the standard case. She defines them by the social function a definition should serve rather than by ordinary usage, on the grounds that the point of the analysis is to guide action, not to catalogue how people already speak [10]. The second is *uptake* as a metric of success: a revised concept starts guiding action only when service configurations, funding, measurement, and communication routines line up behind it. Medicine has done this before. The redefinition of death by neurological criteria did not follow a new discovery; it followed the recognition that the circulatory definition no longer fit what ventilators had made possible, and it went on to reshape statutes, transplant practice, and bedside protocols, though not without decades of dispute about the criteria themselves [11].

Conceptual repair in acute care faces an additional difficulty. U.S. acute care is a complex adaptive system: a network of interacting agents (patients, clinicians, payers, regulators, administrators) whose local incentives and rules generate emergent system‐level behavior, including ED‐as‐default [12, 13]. But concepts are not single levers: “emergency care” enters into laws, payment rules, performance metrics, and clinician training, and coordinates the behavior of many agents at once. Repairing the concept is therefore not a substitute for structural reform; it is the point at which many of the other levers are anchored. Section 4 diagnoses the existing concept and Section 5 reconstructs it along these lines; Section 6 evaluates the result against ethical, psychological, and operational design criteria; and Section 7 addresses uptake.

## 4 Diagnosing the Existing Concept of Emergency Care

Each subsection examines one of the five structures in Figure 1.

**FIGURE 1:**
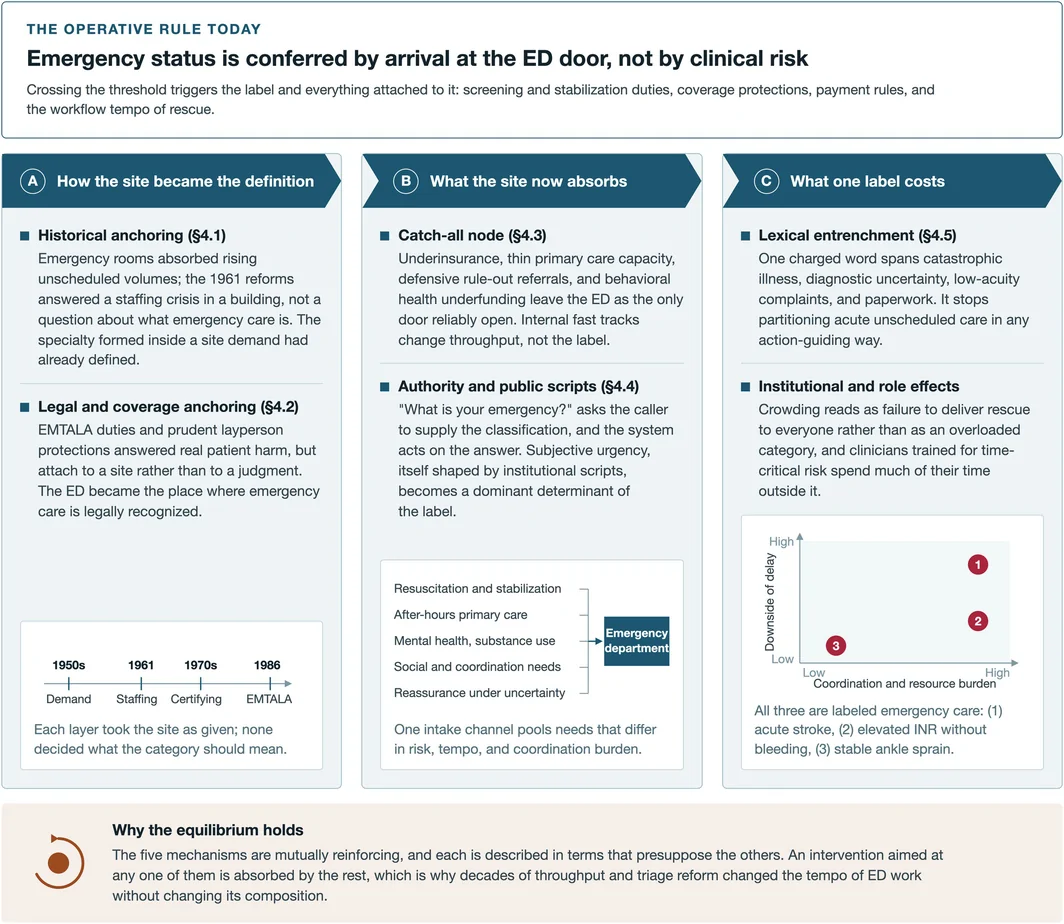
Mechanisms that stabilize the current concept of emergency care. The operative rule today is that emergency status is conferred by arrival at the ED door rather than by clinical risk: Crossing the threshold triggers the label and everything attached to it, including screening and stabilization duties, coverage protections, payment rules, and the workflow tempo of rescue. Panel A shows how history and law located the category in a site. Emergency rooms absorbed rising unscheduled volumes, and the 1961 reforms answered a staffing crisis in a building rather than a question about what emergency care is (Section 4.1); EMTALA duties and prudent layperson protections then answered real patient harm but attached to that site rather than to a clinical judgment (Section 4.2). The inset timeline shows each layer taking the site as given. Panel B shows what the site now absorbs: Underinsurance, thin primary care capacity, defensive rule‐out referrals, and behavioral health underfunding leave the ED as the only door reliably open (Section 4.3), while public scripts ask callers to supply the classification and the system acts on the answer (Section 4.4). The inset shows one intake channel pooling needs that differ in risk, tempo, and coordination burden. Panel C shows what one label costs: A single charged word spans catastrophic illness, diagnostic uncertainty, low‐acuity complaints, and paperwork (Section 4.5), with consequences for institutions and professional roles. The inset case space plots presentations against the downside of delay and the coordination and resource burden they impose: (1) acute stroke, (2) elevated INR without bleeding, and (3) stable ankle sprain, all three of which are currently labeled emergency care. Each mechanism answered a real need or a real harm, which is what makes the arrangement stable; because the five are mutually reinforcing, an intervention aimed at any one of them is absorbed by the rest (Section 4.6).

### 4.1 Historical Anchoring: From Time‐Critical Rescue to Institutional Safety Net

Emergency care developed first in military medicine, organized around a simple insight: when risk is high and time is short, rapid intervention can decisively alter outcomes [14]. But the American institution did not grow from that insight. It grew from demand. Hospital emergency rooms absorbed steeply rising unscheduled volumes through the 1950s, staffed by whoever could be assigned, and the reforms of 1961 (i.e., full‐time physician groups at Alexandria and Pontiac) were answers to a staffing crisis in a building, not to a question about what emergency care is [15]. The specialty was thus constituted inside a site that unscheduled demand had already defined, and formal certification, arriving decades later, ratified an expertise identified by where it was practiced [15]. Prehospital systems might have pulled the other way, and in one sense they did: EMS demonstrated that emergency capability travels [16, 17]. But EMS was federally built after the ED‐based specialty had formed, and it was designed to deliver patients to the ED door rather than to confer emergency status anywhere else. Every subsequent layer took the site as given. No one ever decided that emergency care should mean care delivered in an ED; the identification was the residue of a staffing problem, and the label widened with the building's role until the service was hard to distinguish from the site [18, 19].

### 4.2 Institutional and Legal Anchoring: How Duties and Coverage Consolidate ED‐As‐Default

U.S. professional bodies define emergency medicine as a specialty; none defines emergency care as a service category [19, 20, 21]. Their definitions are nonetheless functional in substance, centering the specialty on immediate decision‐making and action to prevent death or disability through rapid recognition, evaluation, stabilization, and disposition (Table 1). They also stress that the specialty is not tethered to a single site, extending across prehospital response, hospital and freestanding EDs, and urgent care and telemedicine—a claim about the portability of emergency expertise, not an endorsement of extending emergency care to all AUC. But defining the practitioner rather than the service leaves the boundaries of the service unspecified.

**TABLE 1:** Authoritative U.S. definitions relevant to “emergency”: Professional, regulatory, and coverage frames.

Authority (US)	What is being defined	Definition (condensed)	Emphasis/framing
American Board of Medical Specialties (ABMS)	Emergency Medicine (specialty)	A physician specializing in Emergency Medicine focuses on immediate decision‐making and action to prevent death or further disability, including directing prehospital care and providing immediate recognition, evaluation, stabilization, and disposition for acute illness and injury [20]	Professional scope; explicitly spans prehospital + ED, centers on immediacy and stabilization/disposition.
American College of Emergency Physicians (ACEP)	Emergency medicine (where it may be practiced)	ACEP's policy states emergency medicine “may be practiced” in a hospital‐based or freestanding ED, an urgent care clinic, an emergency medical response vehicle, or at a disaster site [21].	Professional, setting‐flexible framing; supports a location‐nonexclusive concept of emergency practice.
EMTALA statute (Social Security Act §1867; 42 U.S.C. §1395dd)	Emergency medical condition (EMC)	An EMC is a condition with acute symptoms of sufficient severity such that lack of immediate medical attention could reasonably be expected to result in serious jeopardy, serious impairment of bodily functions, or serious dysfunction of any bodily organ/part [22]	Risk‐and‐time threshold; anchors legal duties to “immediate medical attention” and serious harm.
CMS EMTALA regulations (42 CFR §489.24)	Dedicated Emergency Department (DED)	A DED is any hospital department/facility (on‐ or off‐campus) that is licensed as an ED, held out to the public as providing urgent emergency care without appointments, or that provides ≥ 1/3 of outpatient visits for treatment of EMCs on an urgent basis [23]	Institution/site trigger for EMTALA obligations; operationalizes what “counts” as an ED.
CMS EMTALA regulations (42 CFR §489.24)	Emergency medical condition (regulatory EMC)	Defines EMC similarly to the statute and explicitly includes acute symptoms (including severe pain) and enumerates serious jeopardy/impairment/dysfunction; explicitly references psychiatric and substance‐use symptoms [23]	Broadens scope in operational terms; supports inclusion of behavioral health and substance‐related emergencies
Federal patient protections for coverage (45 CFR §147.138)	Emergency medical condition (prudent layperson)	An EMC is defined by symptoms such that a prudent layperson could reasonably expect serious jeopardy/impairment/dysfunction without immediate attention [24]	Coverage standard based on symptoms and reasonable belief, not final diagnosis; protects against retrospective denials
Federal patient protections for coverage (45 CFR §147.138)	Emergency services	Emergency services include (A) a hospital ED medical screening exam (and routine ancillary services) and (B) further exam/treatment within hospital capability required to stabilize the patient [24]	Defines what insurers must cover as “emergency services”; ties coverage to screening + stabilization
Medicaid managed care protections (42 CFR §438.114)	EMC & emergency services (Medicaid MCO context)	Defines EMC using the prudent layperson standard; defines emergency services as covered inpatient/outpatient services needed to evaluate or stabilize an EMC [25]	Coverage‐and‐payment rules for Medicaid managed care; reinforces symptom‐based access and stabilization obligations across networks

Regulatory and coverage frameworks, by contrast, operationalize “emergency” through enforceable triggers and entitlements (Table 1), attaching strong consequences to ED presentation [24, 25, 26, 27]. EMTALA imposes screening and stabilization obligations on hospitals with EDs regardless of ability to pay [28], and the prudent layperson standard in commercial insurance and Medicaid managed care defines emergency conditions by symptoms a reasonable non‐clinician could expect to be serious [29]. Both protections answered a real history of patient harm (refusal of care, retrospective denials, and “wallet biopsy” gatekeeping) and neither is the primary source of dysfunction. But both attach their protection to a site rather than to a judgment, and in doing so they converted the ED from the place where emergency care happened into the place where it is legally recognized.

### 4.3 ED as Catch‐All Node and the Social Drivers Behind It

EDs are continuously open, highly visible, and legally constrained in their ability to refuse evaluation. They absorb heterogeneous demands: resuscitation and stabilization, after‐hours primary care substitution, mental health and substance‐use crises, social work needs, and reassurance under uncertainty. Internal segmentation (fast tracks, observation units, triage zones) may improve throughput [30], but institutionally all of it remains emergency care.

The catch‐all function is the predictable result of structural conditions, and the ED's combined clinical and social role is well documented [4, 31]. Underinsurance and uninsurance leave many patients without realistic outpatient alternatives. Primary care capacity has not kept pace with demand, especially in rural and lower‐income settings. Defensive practice generates “rule‐out” referrals into the ED that the referring clinician would prefer not to own. Mental health and substance‐use systems are chronically underfunded, and EDs absorb the overflow. The ED is left with whatever the rest of the system cannot or will not absorb because, in many places, it is the only door reliably open, the only place where prudent layperson protections activate, and the only setting where a clinician can resolve uncertainty without payer interference. None of this is a conceptual failure, and no redefinition repairs it: the conceptual repair proposed here travels with the architectural and access repairs described in Section 7.

### 4.4 Authority and Public Scripts

A distinct distortion concerns who decides when an AUC episode counts as an emergency. Patient‐centered protections such as the prudent layperson standard are ethically important: they acknowledge that patients cannot assess their own risk and guard against punishing those who seek care under reasonable fear [29]. Yet in an ED‐anchored system they function as a patient‐driven quasi‐definition: if a patient arrives with concern that feels urgent, the system treats the encounter as an emergency for screening, coverage, and processing, even when clinical risk proves low [32]. The issue is not irrational patient behavior: given constrained alternatives, the ED is usually the rational choice. The issue is that subjective urgency has become a dominant determinant of what is treated as emergency care—and that urgency is itself shaped by institutional scripts. Dispatch teaches the frame directly: “What is your emergency?” asks the caller to supply the classification, and the system then acts on the answer [33, 34]. Entertainment media supplies the rest, compressing time and implying that definitive testing and rapid resolution are what an ED visit normally delivers [35, 36, 37].

### 4.5 Lexical and Role Effects: One Label, Incompatible Functions

These distortions are amplified by lexical entrenchment. “Emergency” is a charged word: it signals danger, immediacy, and entitlement to rapid mobilization. When the same label is applied across catastrophic illness, moderate‐risk diagnostic uncertainty, low‐acuity complaints, and administrative or social needs, it ceases to partition AUC in an action‐guiding way. It becomes a flattening label, obscuring the differences in risk, time pressure, and resource‐justified priority that ought to drive how care is organized.

This leveling has two downstream effects. First, it creates *institutional incoherence*: because “emergency” connotes maximal urgency, it invites performance regimes and public expectations that treat all ED work as time‐critical. Crowding is then experienced as a failure to deliver an emergency response to everyone, rather than as evidence that the category has been overloaded [38]. Second, it produces *professional role dissonance*. Emergency clinicians are trained around time‐critical risk management, stabilization, and disposition under uncertainty, yet are routinely tasked with large volumes of non‐emergent AUC [39]. The resulting strain is not only fatigue from volume; it is a sustained mismatch between the justificatory narrative of the specialty and the lived content of the work, which is the kind of failure mode that motivates conceptual repair.

### 4.6 Synthesis: Why the Equilibrium Holds

These five structures do not merely coexist; they reinforce one another (Figure 1). The law ratified what history had already built, attaching duties and coverage to a site that unscheduled demand had made central. The catch‐all role gives the public scripts their material, and the scripts return presentations that entrench the catch‐all role. And the label carries the whole arrangement into every statute, contract, and dispatch call, so each part is described in terms that presuppose the others. No one of these structures is a villain. Each answered a real need or a real harm, and that is what makes the equilibrium stable: an intervention aimed at any one of them is absorbed by the other four, which is why decades of throughput and triage reform have changed the tempo of ED work without changing its composition. What the five share is the concept. The next section therefore does not propose to dismantle any of these structures; it re‐specifies the concept they jointly enact.

## 5 Reconstructing Emergency Care Within an Acute Unscheduled Care Ecosystem

The reconstruction has two parts: re‐anchor emergency care in the time‐sensitive risk of serious harm, judged on the information available; and move the conferral of “emergency” status from ED arrival to structured, expert‐mediated triage across a broader AUC network [40].

### 5.1 A Positive Ameliorated Definition

As a guiding characterization: emergency care is a risk‐ and time‐sensitive, expert‐mediated, priority‐conferring subset of acute unscheduled care, conferred by structured triage wherever it occurs. More formally:

Emergency care is the class of interventions and decisions warranted when, on the information available at the time of assessment, appropriately trained clinicians using structured triage judge that a condition carries a substantial risk of serious harm or death within a clinically relevant time horizon, and that this risk justifies priority access to constrained high‐intensity resources.

Three components distinguish this reconstruction. *Risk‐ and time‐sensitivity* reserves emergency care for conditions in which risk and time pressure together justify prioritization, with conservative thresholds when the downside of delay is severe. *Expert mediation* locates the authority to confer the institutional designation in structured triage by trained clinicians rather than in location or self‐designation. Patients' narratives, fears, and values remain central evidence in that judgment; what changes is who holds authority over the label, not whose account counts. *Priority conferral* means the designation activates institutional obligations (i.e., rapid assessment, continuous readiness, escalation capability) that exceed what is owed elsewhere in AUC.

### 5.2 From Location‐Based to System‐Based Triage

Classification becomes the output of a system‐based triage process distributed across multiple entry points (Figure 2), with access protections preserved and explicit escalation pathways when uncertainty remains. In practice, that function runs through several kinds of entry points: clinician‐supported telephone or digital triage; prehospital assessment and routing by EMS; clinician‐staffed community AUC hubs (urgent care, walk‐in centers); primary care and home‐based services; and ED front‐door triage as a high‐capability backstop. What makes this a system rather than a collection of sites is shared thresholds, clear escalation pathways, and documentation that makes triage decisions actionable across settings. A patient with chest pain is risk‐stratified by tele‐triage under shared thresholds, has emergency status conferred at the call, and is routed to EMS with that designation already documented. A patient with an ankle sprain is routed to urgent care with prudent layperson protection attached to the triage decision, not to ED arrival. A patient with a chronic disease flare is connected to home‐based or primary care follow‐up under documented thresholds, with 72‐h returns audited across the network. The anticoagulated patient from the opening example is seen the same day at a community hub or by home‐based services, with point‐of‐care testing, a documented dosing decision, and a scheduled recheck: what the visit actually requires, without the apparatus of rescue. Emergency status is tied to the triage outcome, not to physical arrival.

**FIGURE 2:**
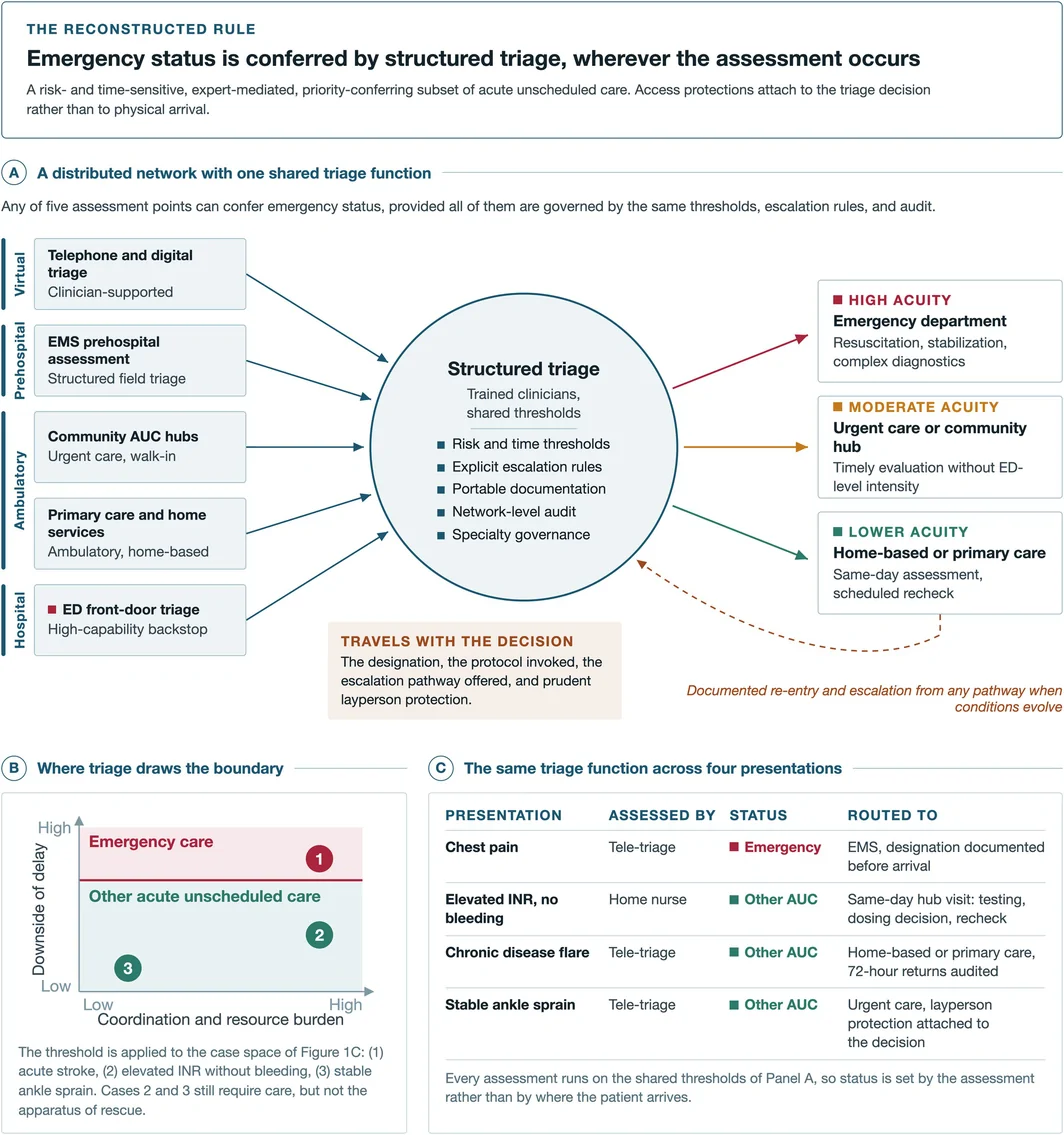
Structured triage as the shared hub of a distributed acute unscheduled care network. The reconstructed rule is that emergency status is conferred by structured triage wherever the assessment occurs, so that access protections attach to the triage decision rather than to physical arrival. Panel A shows the distributed network: Five assessment points, grouped by service area, feed one triage function governed by shared risk and time thresholds, explicit escalation rules, portable documentation, network‐level audit, and specialty governance. That function confers emergency status and routes the episode to one of three coordinated pathways, and the dashed spoke is documented re‐entry and escalation when conditions evolve. ED front‐door triage appears on both sides of the hub because the same site serves as an entry point and as the high‐acuity destination. What travels with the decision is the designation, the protocol invoked, the escalation pathway offered, and prudent layperson protection. Panel B shows the criterion the hub applies, drawn on the case space of Figure 1: Presentations above the threshold on the downside of delay are emergency care, while cases (2) and (3) still require care but not the apparatus of rescue. Panel C traces the same triage function across the four presentations discussed in Section 5.2, showing that status is set by the assessment rather than by where the patient arrives.

### 5.3 Repositioning Emergency Departments Within the Ecosystem

Within this landscape, EDs remain indispensable but are no longer concept‐defining. The ED is one high‐capability node where emergency care is delivered when structured triage confers emergency status or when uncertainty is high enough that ED‐level diagnostic and stabilization capacity is warranted. Triage instruments such as the Emergency Severity Index [41] already operate in this mode within ED walls; the reconstruction extends that logic across the network rather than applying it only after arrival. The payoff is organizational and normative: ED design, staffing, and measurement can align with emergency‐care performance (e.g., timely identification of high‐risk presentations, risk‐adjusted outcomes, safety monitoring for under‐triage) rather than undifferentiated visit volume, and collaboration with lower‐acuity AUC services becomes an explicit coordination problem rather than an implicit definitional expansion.

## 6 Validation: Ethical, Psychological, and Operational Evaluation

The reconstructed concept is offered as an amelioration: a revision intended to better support the ethical and functional work that “emergency care” is asked to perform. The test is whether, under plausible institutional conditions, it supports justified prioritization (ethical), coherent professional roles (psychological), and usable, safe system design (operational). Extended objections and replies are treated in Data S1–S6.

### 6.1 Ethical Evaluation

Tethering emergency care to substantial time‐sensitive risk under reasonable uncertainty offers a public justification for why some cases legitimately claim scarce high‐intensity resources ahead of others [28, 42]. This can strengthen equity insofar as prioritization is anchored in morally relevant stakes rather than contingent factors like arrival mode or capacity to demand attention. The same mechanism carries the proposal's most serious risk: structured triage can encode bias, and routing away from the ED can burden precisely the patients with the fewest alternatives. That is why the measurement bundle in Section 7 stratifies under‐triage by race, language, payer, and geography, and why S.3 treats equity at length. Narrowing emergency care does not imply that lower‐risk AUC lacks ethical standing; it motivates a graded distribution of duties across the ecosystem. The reconstruction does shift classificatory authority from patients to clinicians, and that shift needs defending. It is best defended not by collapsing institutional categories into self‐designation, but by triage that treats patient narratives as central evidence, communicates reasons transparently, and preserves re‐access and escalation when uncertainty remains [43].

### 6.2 Clinician Psychology and Professional Identity

Section 4.5 described the role dissonance the current usage generates [44, 45]. The reconstruction reduces this mismatch by re‐centering emergency care on the cases that ground the specialty's justificatory narrative. Conceptual clarification will not cure burnout; working conditions and staffing remain decisive [46]. But a concept that matches the work emergency clinicians are trained to do removes one persistent source of role confusion.

### 6.3 Operational Considerations

The reconstruction earns its keep only if it can be implemented without increasing avoidable harm. Three constraints follow. First, the *backstop role* of the ED must be preserved for high‐downside uncertainty: when a serious time‐sensitive condition cannot yet be excluded, ED‐level capability is warranted even when its probability is low. Second, routing decisions must be paired with explicit safety‐net instructions and *low‐friction re‐entry* *escalation pathways*. Third, triage must be treated as a *system function with accountability*: thresholds conservative where diagnostic capacity is limited, outcomes monitored across the AUC network, and patterns of under‐triage triggering protocol revision. Whether these mechanisms yield net benefit is an empirical question, not one a definition can settle.

## 7 Practical Steps Forward

The uptake condition from Section 3 sets the agenda here. The aim is not to redesign U.S. acute care from first principles but to identify what can be started inside a single department within a year, whatever its setting, scaled regionally, and pursued at the policy level over a decade, and to be explicit about which settings can attempt which steps (Section 7.2).

### 7.1 A Phased Minimum Viable Pathway

Table 2 sets out a pathway across three time horizons: the first tier changes what a department knows, the second what a region does, and the third the rules that hold the current equilibrium in place. The 0–12 month tier is deliberately modest. It builds governance and measurement and changes no patient's path: routing protocols run in shadow mode, with the triage recommendation documented and audited but not enforced, so the system learns its own under‐ and over‐triage rates before redirection outruns the evidence for its safety. In shadow mode, under‐triage is read from the documented recommendation against the patient's actual course (a non‐ED recommendation followed by admission or a significant finding); rerouting‐based signals such as 72‐h ED returns apply once routing goes live.

**TABLE 2:** A phased minimum viable pathway for reengineering the conferral architecture.

Tier	What a delivery system can do (academic center example)	Required capabilities	Risks to manage
0–12 months (start now)	Stand up a clinician‐led AUC steering group (EM + primary care + EMS + urgent care + informatics + nursing + community/equity lead). Define 5–8 syndrome‐specific routing protocols (e.g., chest pain, abdominal pain, dyspnea, infant fever, dizziness in older adults, mental health crisis, wound issues, anticoagulation concerns) with explicit green/yellow/red logic and an EM‐attending backup rule. Build a measurement bundle: under‐triage signals (e.g., 72‐h ED return with significant findings), equity stratification, payer denial monitoring, patient trust. Run routing protocols in shadow mode for the first year: the recommendation is documented and audited, not enforced	Existing ED leadership, primary care relationships, modest informatics support, EHR documentation conventions that record triage decisions and prudent layperson protections	Under‐triage of older adults and patients with diagnostic disparities; perceived paternalism if routing is opaque; payer denial of routed‐away care
1–3 years	Embed triage logic into the EHR and patient portal with human‐in‐the‐loop oversight; develop a tele‐triage clinical workforce model with clear EM oversight; realign hospital metrics away from “ED volume reduction” and toward time‐sensitive outcome protection plus safe routing; expand syndrome coverage and audit cadence; integrate with community AUC partners	Informatics and clinical decision support investment; tele‐triage credentialing and workflow; service‐line cooperation; payer engagement on tele‐triage and AUC reimbursement	EHR‐mediated bias amplification; rural and digital‐access inequities; metric gaming if old volume incentives are not fully retired
3–10 years	Regionalize high‐acuity emergency care capacity; build accountable AUC hubs with shared thresholds and joint audit; pursue payment reform that funds non‐ED AUC and tele‐triage; extend EMTALA‐equivalent screening/stabilization protections to designated AUC hubs and tele‐triage services; align ABEM/ACEP/SAEM/AACEM workforce policy around system‐level acute risk governance	Federal and state regulatory engagement; payer realignment; specialty‐society coordination; sustained infrastructure investment in non‐ED AUC, especially in underserved geographies	Fragmentation of the safety net if AUC hubs are unevenly distributed; regulatory unintended consequences; loss of integrative ED role if not preserved by design

Payment here deserves separate emphasis. Under fee‐for‐service payment, ED visits and the admissions that flow from them are often financially favorable for hospitals, so redirecting patients away from the ED can run against the financial interest of the organization asked to lead the change [47]. The pathway is sequenced with this constraint in mind. The first tier requires no revenue shift. The later tiers depend on payment models that reward total‐cost and outcome performance rather than visit volume. Systems already bearing that kind of financial risk, through global budgets, capitation, accountable care contracts, Medicare Advantage, or integrated payer‐provider arrangements, are the natural early adopters. Where no such model exists, the honest conclusion is that the later tiers wait on payment reform, not that the concept fails.

### 7.2 Applying the Pathway Across Delivery Settings

The pathway presumes, at its fullest, a degree of integration (cooperative EMS, interoperable records, aligned payers) that few U.S. regions possess. The reconstructed concept does not require every setting to build the same system. It requires each setting to answer the same question: where can structured, accountable triage actually happen, and what follows when the answer is “only at the ED”? Table 3 summarizes what the concept implies across five delivery settings, the minimum preconditions of each application, and the failure mode each should watch.

**TABLE 3:** Applying the reconstructed concept across delivery settings.

Setting	What the concept implies	Minimum preconditions	Primary failure mode to monitor
Integrated delivery system/payer‐provider organization	Full pathway (Table 2), including cross‐setting triage with shared thresholds and network‐level audit	Tele‐triage capacity; owned or contracted non‐ED AUC sites; total‐cost financial risk; unified governance	Under‐triage driven by cost incentives; erosion of the ED backstop role
Academic center in a fragmented market	Tier 1 governance and measurement; shadow‐mode routing protocols; partnerships (EMS, urgent care) added as they can be negotiated	ED and primary care leadership commitment; modest informatics support; at least one willing external partner	Protocols that exist on paper but do not change practice; partner turnover stalling governance
Community (non‐teaching) hospital	Tier 1 measurement bundle; adoption of externally validated routing protocols rather than local development; regional escalation agreements	Medical staff engagement; access to a regional protocol library and audit support	Under‐resourced audit; protocol drift without local adaptation
Rural/critical access hospital	Designate the ED as the community's AUC hub; bring tele‐triage and specialty support into it from regional centers; payment sufficient to sustain the hub role	Reliable connectivity; transfer agreements; a regional hub willing to supply consultation and joint audit	Loss of the only local access point if payment lags the designation; transfer delays for time‐critical patients
Safety‐net/public system	Equity‐first sequencing: measurement and community co‐design before any routing change; walk‐in access preserved throughout	Community and patient participation in design; stable funding for non‐ED AUC before any diversion begins	Routing that functions as a barrier for the patients with the fewest alternatives

Integrated delivery systems and payer‐provider organizations already operate tele‐triage, own primary and urgent care capacity, and bear financial risk for total cost of care; for them the full pathway is a near‐term possibility, and they are where the empirical case for or against the reconstruction can be made first. Rural and critical access settings sit at the opposite pole, and there the concept's implication reverses. Where there is no urgent care, no home‐based acute care, and thin telehealth, routing patients away from the ED is neither possible nor appropriate. Applying the concept means formally designating the rural ED as the community's hub for all acute unscheduled care, resourcing and paying it for that role, and extending tele‐triage and specialty support into it from regional centers. For rural emergency care, the reconstruction is an argument for investment, not for bypass.

Even where every precondition is absent, the concept still does useful work inside a single ED. Internal streams can be designed, staffed, and measured as emergency care and as other AUC, so that performance expectations match the work being done and under‐triage between streams is audited as it would be across a network. This most modest application is available to every ED regardless of market structure; it is the seed from which the system‐level versions grow.

### 7.3 Workforce: Where Emergency‐Medicine‐Trained Clinicians Fit

For the specialty, the central question is what becomes of emergency medicine in a decentralized model. Its identity shifts from staffing a node to governing a system. Three claims orient that shift. First, *emergency medicine expertise remains indispensable* for the cases the reconstructed concept identifies as emergency care: high‐downside uncertainty, resuscitation and stabilization, escalation under time pressure, and governance of the triage thresholds that determine which patients reach ED‐level care. Second, *not every AUC routing decision requires an emergency physician*. Tele‐triage, urgent care, EMS routing, and home‐based AUC can be staffed by registered nurses, advanced practice providers, paramedics, and primary care clinicians operating within structured protocols and explicit escalation rules. What emergency medicine should own is the *standards*, *audit*, and *failure‐mode review* across the AUC network. That governance role must also fit hospitals with no residency‐trained emergency physician on site, the reality in many rural and critical access EDs, where it takes the form of regional support (tele‐consultation, transfer agreements, shared protocols, and joint audit) supplied by better‐resourced hubs rather than on‐site specialty staffing. Third, *specialty leadership must pivot*. ABEM, ACEP, SAEM, and AACEM have a near‐term opportunity to reposition emergency medicine from “the specialty that staffs EDs” to “the specialty that governs system‐level acute risk.” If that pivot does not happen, jurisdiction over routing will be ceded to payers, retail health, and algorithmic triage products already moving into the space.

### 7.4 A Worked Example: The First Year at One Center

The example below is drawn from an academic center, not because the pathway requires one, but because Tier 1 was designed to be intra‐organizational, and academic centers can run a first year with research‐grade audit and publish the results. Table 3 states what the same tier looks like elsewhere.

*Governance*. A clinician‐led AUC steering group co‐chaired by emergency medicine and primary care, with EMS, urgent care, informatics, nursing leadership, and a community/equity lead. The group owns triage thresholds, escalation rules, and audit.

*Routing*. Five to eight syndrome‐specific protocols (e.g., chest pain, dizziness in older adults, anticoagulation concerns; Table 2 lists the full set) with explicit green/yellow/red routing logic and an emergency‐medicine‐attending backup rule for any uncertain case.

*Escalation*. Default‐to‐ED rules for uncertainty, scheduled callbacks for routed‐away patients, and “return anytime” language with documented prudent layperson protection.

*Measurement*. A standing dashboard tracking under‐triage signals (72‐h ED returns with significant findings), equity stratification by race, language, payer, and geography, payer denial monitoring, and patient trust/satisfaction. The dashboard reports to the steering group monthly.

*Payer protection*. Documentation conventions recording the triage recommendation, the protocol invoked, and the escalation pathway offered so retrospective denial for patients who follow triage advice is identifiable and contestable.

Even one well‐specified configuration of this kind, run for a year and audited honestly, would show whether the reconstructed concept is implementable and where it needs adjustment.

## 8 Conclusion

The concept of emergency care in many U.S. health systems is overloaded and location‐bound: a category built for battlefield rescue, left to absorb whatever unscheduled demand arrived at the one door always open. Using conceptual engineering, I proposed a replacement: emergency care as a risk‐ and time‐sensitive, expert‐mediated, priority‐conferring subset of AUC, conferred by structured triage wherever it occurs. What a setting can attempt depends on its integration, payment model, and alternatives to the ED; in the least‐resourced settings the concept argues for strengthening the ED as the local acute care hub rather than routing around it. The near‐term test is empirical, and Section 7.4 describes it: one department, one year, shadow‐mode protocols, honest audit. The longer test is jurisdictional: whether emergency medicine takes ownership of the standards, audit, and governance of AUC routing, or cedes that ground to payers, retail health, and algorithmic triage. The concept will be repaired either way. The only question is by whom.

## Author Contributions

R.A.T. conceived, wrote, and takes responsibility for the manuscript as a whole.

## Conflicts of Interest

The author declares no conflicts of interest.

## Supporting information


**Data S1:** Hypothetical objections and replies.

## Data Availability

The author has nothing to report.

## References

[1] A. L. Kellermann , R. Y. Hsia , C. Yeh , and K. G. Morganti , “Emergency Care: Then, Now, and Next,” Health Aff (Millwood) 32, no. 12 (2013): 2069–2074. 10.1377/hlthaff.2013.0683 24301388

[2] R. M. Weinick , R. M. Burns , and A. Mehrotra , “Many Emergency Department Visits Could Be Managed at Urgent Care Centers and Retail Clinics,” Health Aff (Millwood) 29, no. 9 (2010): 1630–1636. 10.1377/hlthaff.2009.0748 PMC3412873 20820018

[3] A.‐C. Durand , S. Gentile , B. Devictor , et al., “ED Patients: How Nonurgent Are They? Systematic Review of the Emergency Medicine Literature,” American Journal of Emergency Medicine 29, no. 3 (2011): 333–345. 10.1016/j.ajem.2010.01.003 20825838

[4] J. A. Gordon , “The Hospital Emergency Department as a Social Welfare Institution,” Annals of Emergency Medicine 33, no. 3 (1999): 321–325. 10.1016/s0196-0644(99)70369-0 10036347

[5] B. R. Asplin , “Tying a Knot in the Unraveling Health Care Safety Net,” Academic Emergency Medicine 8, no. 11 (2001): 1075–1079. 10.1111/j.1553-2712.2001.tb01118.x 11691671

[6] G. Löhr and H. Veluwenkamp , “Rethinking Philosophical Methodology: Conceptual Engineering Meets Value Sensitive Design,” Metaphilosophy 56, no. 3–4 (2025): 328–343.

[7] M. Simion and C. Kelp , Knowledge and Conceptual Engineering (Oxford University Press, 2026).

[8] D. J. Chalmers , “What Is Conceptual Engineering and What Should It Be?,” Inquiry 68, no. 9 (2025): 2902–2919.

[9] H. Cappelen , “Conceptual Engineering,” in Conceptual Engineering and Conceptual Ethics (Oxford University Press, 2020), 132–151.

[10] S. Haslanger , “Going on, Not in the Same Way,” in Conceptual Engineering and Conceptual Ethics (Oxford University Press, 2020), 230–260.

[11] Ad Hoc Committee of the Harvard Medical School to Examine the Definition of Brain Death , “A Definition of Irreversible Coma,” JAMA 205, no. 6 (1968): 337–340. 5694976

[12] P. E. Plsek and T. Greenhalgh , “Complexity Science: The Challenge of Complexity in Health Care,” BMJ 323, no. 7313 (2001): 625–628. 10.1136/bmj.323.7313.625 PMC1121189 11557716

[13] D. H. Meadows , Leverage Points: Places to Intervene in a System (Sustainability Institute, 1999).

[14] B. J. Zink , Anyone, Anything, Anytime: A History of Emergency Medicine (Mosby, 2005).

[15] Institute of Medicine , Hospital‐Based Emergency Care: At the Breaking Point (National Academies Press, 2007).

[16] R. Mackenzie , “Brief History of Pre‐Hospital Emergency Medicine,” Emergency Medicine Journal 35, no. 3 (2018): 146–148. 10.1136/emermed-2017-207310 29463631

[17] C. A. Rockwood, Jr. , C. M. Mann , J. D. Farrington , O. P. Hampton, Jr. , and R. E. Motley , “History of Emergency Medical Services in the United States,” Journal of Trauma 16, no. 4 (1976): 299–308. 10.1097/00005373-197604000-00008 772229

[18] B. J. Yun , M. K. Singh , M. A. Reznek , et al., “Strengthening Essential Emergency Departments: Transforming the Safety Net,” Health Affairs Scholar 3, no. 3 (2025): qxaf044. 10.1093/haschl/qxaf044 PMC11904886 40084221

[19] S. M. Schneider , G. C. Hamilton , P. Moyer , and J. S. Stapczynski , “Definition of Emergency Medicine,” Academic Emergency Medicine 5, no. 4 (1998): 348–351. 10.1111/j.1553-2712.1998.tb02720.x 9562202

[20] American College of Emergency Physicians , “Definition of Emergency Medicine,” Annals of Emergency Medicine 52, no. 2 (2008): 189–190. 10.1016/j.annemergmed.2008.05.029 18655932

[21] Institute of Medicine , Emergency Medical Services: At the Crossroads. Future of Emergency Care (National Academies Press, 2007).

[22] American Board of Emergency Medicine , “American Board of Medical Specialties,” (2026).

[23] American College of Emergency Physicians , “Freestanding Emergency Departments and Urgent Care Centers Information Paper,” (2026).

[24] 42 U.S. Code §1395dd , “Examination and Treatment for Emergency Medical Conditions and Women in Labor,” (1986).

[25] 42 CFR §489.24 , “Special Responsibilities of Medicare Hospitals in Emergency Cases,” (1986).

[26] 45 CFR §147.138 , “Patient Protections,” (2026).

[27] 42 CFR §438.114 , “Emergency and Post‐Stabilization Services,” (2002).

[28] R. A. Bitterman , “EMTALA and the Ethical Delivery of Hospital Emergency Services,” Emergency Medicine Clinics of North America 24, no. 3 (2006): 557–577. 10.1016/j.emc.2006.05.003 16877130

[29] J. Li , H. K. Galvin , and S. C. Johnson , “The “Prudent Layperson” Definition of an Emergency Medical Condition,” American Journal of Emergency Medicine 20, no. 1 (2002): 10–13. 10.1053/ajem.2002.30108 11781904

[30] B.‐J. Lowie , C. Hicks , C. Falat , et al., “Overcoming Stagnant Flow: A Scoping Review of Vertical Movement in the Emergency Department,” Academic Emergency Medicine 31, no. 3 (2024): 256–262. 10.1111/acem.14846 38060332

[31] E. S. Anderson , D. Hsieh , and H. J. Alter , “Social Emergency Medicine: Embracing the Dual Role of the Emergency Department in Acute Care and Population Health,” Annals of Emergency Medicine 68, no. 1 (2016): 21–25. 10.1016/j.annemergmed.2016.01.005 26921967

[32] A. Morgans and S. J. Burgess , “What Is a Health Emergency? The Difference in Definition and Understanding Between Patients and Health Professionals,” Australian Health Review 35, no. 3 (2011): 284–289. 10.1071/AH10922 21871188

[33] E. Ellcessor , In Case of Emergency: How Technologies Mediate Crisis and Normalize Inequality (NYU Press, 2022).

[34] K. Johnson , “Crisis Communication: What is Your Emergency?,” (2013).

[35] E. B. Tapper , “Doctors on Display: The Evolution of Television's Doctors,” Proceedings (Baylor University Medical Center) 23, no. 4 (2010): 393–399. 10.1080/08998280.2010.11928659 PMC2943455 20944763

[36] D. Harris and H. Willoughby , “Resuscitation on Television: Realistic or Ridiculous? A Quantitative Observational Analysis of the Portrayal of Cardiopulmonary Resuscitation in Television Medical Drama,” Resuscitation 80, no. 11 (2009): 1275–1279. 10.1016/j.resuscitation.2009.07.008 19699021

[37] B. A. Primack , T. Roberts , M. J. Fine , F. R. Dillman Carpentier , K. R. Rice , and A. E. Barnato , “ER vs. ED: A Comparison of Televised and Real‐Life Emergency Medicine,” Journal of Emergency Medicine 43, no. 6 (2012): 1160–1166. 10.1016/j.jemermed.2011.11.002 PMC3573875 22766407

[38] G. D. Kelen , R. Wolfe , G. D'Onofrio , et al., “Emergency Department Crowding: The Canary in the Health Care System,” NEJM Catalyst 2, no. 5 (2021): 217.

[39] E. Harmon‐Jones and J. Mills , An Introduction to Cognitive Dissonance Theory and an Overview of Current Perspectives on the Theory (American Psychological Association, 2019).

[40] S. Aikin , “Conceptual Engineering, Amelioration, and Not Changing the Subject,” Southwest Philosophy Review 41, no. 2 (2025): 17–21.

[41] N. Gilboy , P. Tanabe , D. Travers , and A. M. Rosenau , Emergency Severity Index (ESI): A Triage Tool for Emergency Department Care, Version 4. Implementation Handbook 2012 Edition (Agency for Healthcare Research and Quality, 2011).

[42] G. Bognar , “A Theory of Triage,” Bioethics 38, no. 2 (2024): 95–106. 10.1111/bioe.13242 37991489

[43] N. Davoudi , N. D. Nayeri , M. S. Zokaei , N. Fazeli , and P. F. Carspecken , “Culture of Paternalism in the Emergency Department: A Critical Ethnographic Study,” BMC Health Services Research 25, no. 1 (2025): 1068. 10.1186/s12913-025-13282-8 PMC12344830 40797195

[44] C. Schrepel , J. Jauregui , A. Brown , J. Shandro , and J. Strote , “Navigating Cognitive Dissonance: A Qualitative Content Analysis Exploring Medical Students' Experiences of Moral Distress in the Emergency Department,” AEM Education Training 3, no. 4 (2019): 331–339. 10.1002/aet2.10380 PMC6795361 31637350

[45] C. Meyer , C. Chiapponi , F. von Kaufmann , K.‐G. Kanz , and D. Hinzmann , “Occupational Stress Profiles of Prehospital and Clinical Staff in Emergency Medicine: A Cross‐Sectional Baseline Study,” Frontiers in Public Health 12 (2024): 1480643. 10.3389/fpubh.2024.1480643 PMC11472573 39403439

[46] A. Jachmann , A. Loser , A. Mettler , A. Exadaktylos , M. Müller , and K. Klingberg , “Burnout, Depression, and Stress in Emergency Department Nurses and Physicians and the Impact on Private and Work Life: A Systematic Review,” Journal of American College of Emergency Physicians Open 6, no. 2 (2025): 100046. 10.1016/j.acepjo.2025.100046 PMC11872394 40026616

[47] D. A. Handel , J. A. Hilton , M. J. Ward , E. Rabin , F. L. Zwemer, Jr. , and J. M. Pines , “Emergency Department Throughput, Crowding, and Financial Outcomes for Hospitals,” Academic Emergency Medicine 17, no. 8 (2010): 840–847. 10.1111/j.1553-2712.2010.00814.x 20670321

