# Supplementary material for: Reengineering the Concept of Emergency Care
Source: Acad Emerg Med. 2026 Oct 9;33(10):e70424. doi: 10.1111/acem.70424 (PMC13651702; doi:10.1111/acem.70424)
Supplement: Supplementary file 1 [file ACEM-33-0-s001.docx]

**Supplement**

*Hypothetical objections and replies*

**Introduction to the Supplement**

The conceptual proposal in the main text invites a number of reasonable objections. The objections and replies collected here were developed during the conceptual analysis as deliberate stress tests of the proposal. Each names a way the reconstruction could plausibly fail in practice, and each reply either revises the claim, narrows its scope, or identifies the institutional conditions under which the worry is genuinely binding. The aim is not to dismiss the objections but to make the limits and prerequisites of the proposal explicit, so that any attempt at uptake can confront the same questions before, rather than after, deployment.

**S.1 Risk of exclusion and missed emergencies**

**Objection.** Narrowing the extension of emergency care and externalizing triage may increase the risk that genuine emergencies are misclassified as non-emergent and routed away from ED-level services. Any additional triage layer in a strained system risks becoming a barrier to care, especially for patients with atypical presentations or limited ability to advocate for themselves. The current over-inclusive concept (“if you make it to the ED, you count”) functions as a safety net; tightening conceptual and institutional thresholds may compromise that safety.

**Reply.** This is the most important objection, and the reconstructed concept is defensible only if paired with explicit protective design. Three safeguards are non-optional. First, triage must default to ED routing under uncertainty, especially in low-resource settings and in populations with documented diagnostic disparities; the proposal’s “backstop” role for the ED is therefore a constitutive feature, not a concession. Second, every routed-away patient must receive a low-friction re-entry pathway, including explicit return instructions, scheduled callbacks, and preserved prudent layperson protections, so that evolving presentations can be reclassified without penalty or delay. Third, under-triage signals (most operationally, rerouted patients who arrive at an ED within 72 hours with significant findings) must be audited routinely and trigger protocol revision and, where necessary, local relaxation of thresholds. The reconstruction does not claim that fewer people deserve care; it claims that emergency care status should track risk under uncertainty rather than location of arrival. Where the empirical reality is that this cannot be done safely for a particular population or setting, the appropriate response is to widen the threshold locally, not to abandon the concept. Conceptual engineering here is not a license for rigidity; it is a framework for making protective design choices explicit and revisable.

**S.2 Paternalism and marginalizing lay perspectives**

**Objection.** By anchoring the classification of emergencies in expert-mediated triage rather than in patient self-designation or a prudent layperson standard, the reconstructed concept may appear paternalistic. It could be read as saying that patients are mistaken about whether they are in an emergency and that only professionals can determine the “true” status of their condition. This risks discounting patient experience, undermining trust, and reproducing historical patterns in which marginalized groups’ accounts of their symptoms are systematically devalued.

**Reply.** The proposal distinguishes between the subjective experience of emergency, which can be ethically weighty even when clinical risk is low (e.g., a frightened caregiver with a febrile child, a person in acute psychological distress), and the institutional category of emergency care, which must organize priority access to scarce resources across a population. Anchoring the latter in expert-mediated triage does not entail that patient perspectives are irrelevant; the reconstructed concept presupposes that triage is conducted in dialogue with the patient, with clinicians relying on patients’ narratives, contextual knowledge, and values to make risk assessments. What changes is not whose voice matters, but what classification is being made and for what purpose. The current configuration is also not value-neutral. Treating any ED arrival as an “emergency” for institutional purposes does not actually give patients more authority; it ties their fate to where they happen to present and to opaque back-end processes they cannot see. A system-based triage model, if designed transparently, can be more respectful: it can tell patients honestly, “We take your concern seriously; here is why we think this level of care is appropriate now, and here is how you can escalate if things change.” The paternalism objection nonetheless points to the need for participatory design of triage criteria and AUC pathways (including patient and community voices), communication training, and ongoing evaluation of whether certain groups feel systematically diverted. Without those elements, the reconstructed concept could be implemented in a paternalistic way; the concept does not guarantee good practice, but it can be used to argue for it.

**S.3 Reinforcing or reconfiguring inequities**

**Objection.** Even with the best intentions, system-based triage and diversified AUC pathways may reproduce or amplify inequities. Populations with less digital access may be effectively excluded from tele-triage and virtual AUC options; algorithmic or protocol-driven triage may encode biases that under-estimate risk in certain demographic groups; and “alternative pathways” may, in practice, be of lower quality in under-resourced communities, turning the conceptual distinction between emergency care and AUC into a de facto segregation of services. The current overreliance on EDs, while inefficient, at least offers a relatively uniform entry point. Fragmentation of AUC could lead to unevenness in who actually reaches emergency care when needed.

**Reply.** This objection points to a real danger. The reconstructed concept is ethically acceptable only if implemented within an AUC system designed with equity as a central constraint, not an afterthought. That implies several commitments. Triaged pathways must be comparable in quality across communities; if certain regions lack safe non-ED AUC, routing away from the ED there is inappropriate. In rural settings, the appropriate application is the one described in §7.2 of the main text: designate and resource the ED itself as the community’s AUC hub, rather than routing around it. Triage tools should be validated and recalibrated with explicit attention to performance across racial, socioeconomic, and linguistic groups. Equity metrics (stratified rerouting outcomes, payer denial rates, patient trust) must be built into the governance of the AUC system from day one. Importantly, the present concept of emergency care does not avoid inequity; it hides it behind ED doors. Patterns of delayed presentation, differential pain assessment, and variable admission thresholds already produce inequitable emergency care. The reconstructed concept, by making classifications and pathways more explicit, can expose inequities to view and thereby make them more corrigible. Whether that potential is realized depends on political and institutional will, not on conceptual refinement alone.

**S.4 Implementation infeasibility and “conceptual idealism”**

**Objection.** The proposal may seem utopian. Many health systems struggle to maintain basic ED staffing and inpatient capacity; building tele-triage, urgent care networks, home-based AUC, and accountable triage governance might appear so far beyond current capabilities that the reconstructed concept feels like an exercise in conceptual idealism: attractive in theory, irrelevant in practice.

**Reply.** The objection mislocates the role of the conceptual project. The argument is not that systems can instantiate an ideal AUC ecosystem by definitional fiat; it is that without a clarified concept of emergency care, it is difficult even to correctly diagnose current dysfunctions or set coherent targets for incremental reform. A system might experiment with tele-triage or home-based acute care but evaluate success solely as ED visit reduction, without distinguishing visits that are conceptually appropriate to avoid from those that represent failures of access to emergency care. The reconstructed concept supplies a normative compass: the goal is not “less ED use” but a rebalanced AUC system in which emergency care is deployed where it matters most. Implementation will be heterogeneous and gradual: modest tele-triage pilots for selected complaints, regional urgent care partnerships, subpopulation-focused programs for high-risk chronic disease, or internal language and metric revisions before larger structural changes. The conceptual proposal provides a direction of travel, and the minimum viable pathway in Table 2 of the main text translates that direction into actions a delivery system can begin within twelve months, while §7.2 and Table 3 specify how the pathway scales down, or reverses direction, across integrated, community, rural, and safety-net settings. Where regional integration is absent, the pathway does not presume it into existence: the first-year tier is deliberately intra-organizational, and routing changes wait on the preconditions Table 3 makes explicit. Recognizing practical constraints does not undercut the value of having a clearer sense of what is being built.

**S.5 Misdiagnosis: “the problem is capacity, not concepts”**

**Objection.** A more fundamental critique is that the paper misdiagnoses the problem. On this view, the main drivers of ED crowding, burnout, and suboptimal care are bluntly material: insufficient inpatient beds, inadequate primary care access, underfunded mental health services, and rising demand. The concept of emergency care may be messy, but changing it will not conjure beds, staff, or community resources. Conceptual engineering risks distracting from the real work of capacity building and policy reform.

**Reply.** Capacity constraints are real drivers of dysfunction, and the main text concedes this explicitly. The argument is not that conceptual ambiguity is the sole or even primary cause of current crises, but that it is a non-trivial contributor and, more importantly, a lever for shaping policy responses. Concepts structure how problems are seen and which solutions count as legitimate. If all ED use is treated as undifferentiated emergency care, EDs are normalized as the default site for unmet needs, reforms focus on increasing ED capacity rather than diversifying AUC options, and clinicians’ alienation gets dismissed as resistance to “what the job has become.” By contrast, a re-engineered concept that distinguishes emergency care from AUC justifies investments in non-ED AUC as enabling rather than diluting emergency care, supports the policy claim that ED crowding is partly a sign of conceptual and structural misalignment rather than bad luck, and legitimizes clinical and administrative efforts to redesign pathways around risk and time-sensitivity. Conceptual engineering does not replace material reforms; it guides them. Treating conceptual work as sufficient would be misdiagnosis. Treating it as necessary but incomplete is the position of this paper.

**S.6 Loss of the ED’s integrative safety-net role**

**Objection.** Clarifying the concept of emergency care and building alternative AUC pathways could erode the ED’s role as an integrative safety net. EDs are often the only place where patients’ complex medical and social needs are seen in their full interdependence. Fragmenting unscheduled care into multiple sites and services risks shunting patients between silos, with no one responsible for seeing the whole picture. The ED’s very over-inclusiveness may be a feature, not a bug, in systems characterized by fragmentation.

**Reply.** The worry highlights a genuine value of EDs as they currently function: they are one of the few remaining points of convergence in many systems. The reconstructed concept does not deny this value; it questions whether the concept “emergency care” should be coextensive with this safety-net function. Two points are important. First, the integrative role can be acknowledged and preserved without labeling all functions performed in EDs as emergency care. An ED might house emergency teams, AUC services, social work and case management, and psychiatric crisis response under one roof; conceptually only some of this is emergency care, while institutionally the ED still functions as a convergence point. Second, a well-designed AUC ecosystem can create multiple complementary integration points (community hubs that combine urgent clinical care, mental health support, and social services). The goal is not to strip EDs of their safety-net role but to ensure they are not the only site where integration occurs, and that emergency care is not overloaded with responsibilities better handled elsewhere. If attempts to diversify AUC produce further fragmentation, that counts against the way the concept has been implemented, not against the conceptual distinction itself. The objection thus reinforces the need to design AUC not as a scattered set of “alternatives to the ED” but as a coordinated network with clear responsibility for whole-person assessment and follow-up.
